# Supplementary material for: Elucidation of the aberrant 3′ splice site selection by cancer-associated mutations on the U2AF1
Source: Nat Commun. 2020 Sep 21;11:4744. doi: 10.1038/s41467-020-18559-6 (PMC7505975; doi:10.1038/s41467-020-18559-6)
Supplement: Supplementary file 1 — Supplementary Information [file 41467_2020_18559_MOESM1_ESM.pdf]

Supplementary Information

Elucidation of the aberrant 3´ splice site selection by cancer-associated mutations on the U2AF1

Hisashi Yoshida et al.

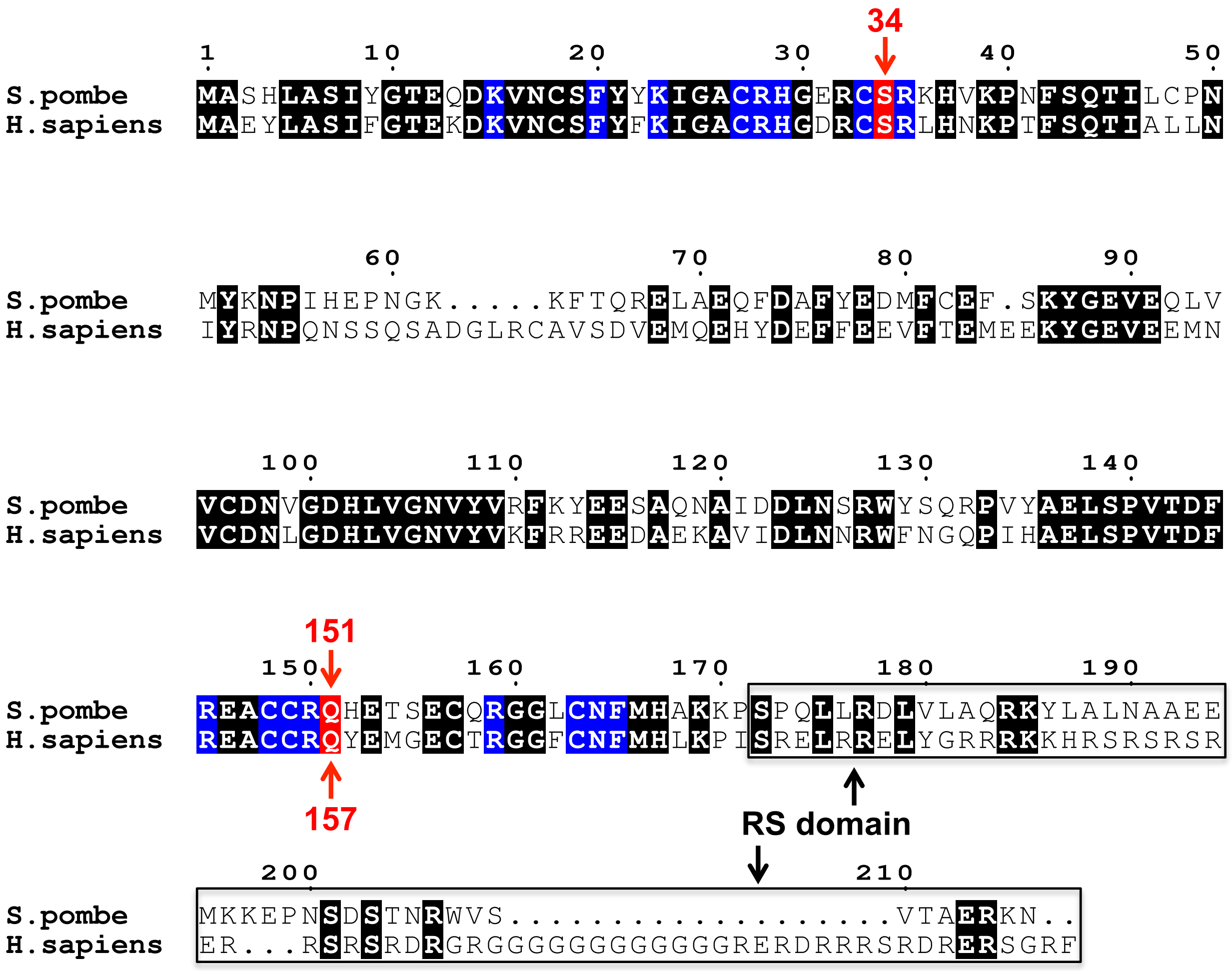

Supplementary Fig. 1

The sequence alignment of *S.pombe* U2AF1 with that from *H.sapiens*.

Sequence alignment was carried out by *Clustal Omega*[1], and the figure was produced by *ESPript* 3.0[2]. Numbering is based on the *S.pombe* U2AF1. Residues blocked in black are conserved, and residues blocked in blue interact with RNA. Residues of S34 and Q151 (Q157 in *H.sapiens*) which are pathogenic mutant of U2AF1, are blocked in red. C-terminus of RS domain (178-240 in *H.sapiens*) is enclosed with solid line. The GeneBank accession numbers are as follows: *S.pombe*, Q09176 and *H.sapiens*, Q01081.

a

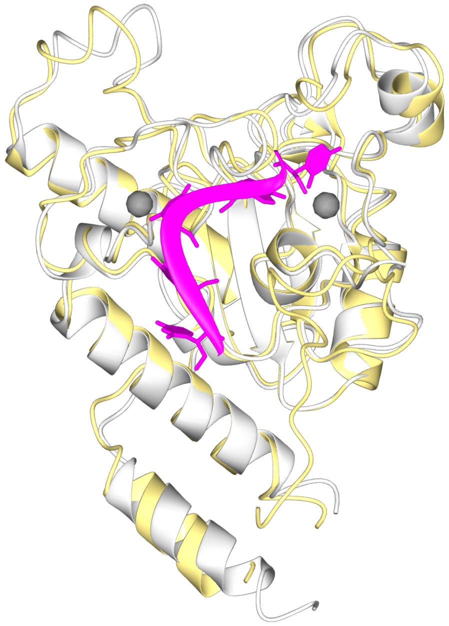

b

chain	rmsd (Å)	Number of residues
A	0.79	178
D	0.59	177
G	0.98	175
J	0.77	178
M	0.68	178
P	0.90	175
S	0.64	177
V	0.93	174
Y	0.93	177

c

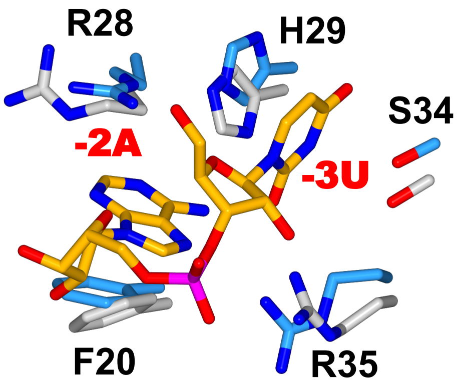

d

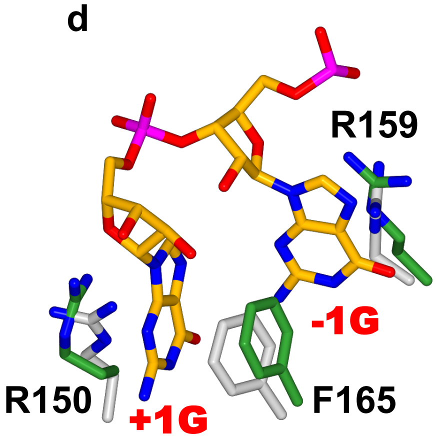

Supplementary Fig. 2

Structural comparison between RNA-free and RNA-bound states of U2AF1.

(a) Overlay of the overall U2AF1 structure (chain-A) with RNA (UUAGG) and that without RNA (PDB ID 4YH8) [3]. The two structures are represented as ribbon models. Protein structure of RNA-free form is shown in light grey, whereas that of RNA-bound form is in yellow. RNA in RNA-bound form is colored in magenta. Structural alignment was carried out automatically with SSM (Secondary Structure Matching) through *COOT* [4]. **(b)** The Cα rmsd, calculated for each U2AF1 molecule in the asymmetric unit, comparing the models with and without bound RNA. The number of atoms used in each calculation is shown. **(c)** Close-up view of (a) for the RNA binding site of the N-terminal zinc finger (ZF1, 2-43). In the RNA-bound form, protein and

RNA are shown in stick representation colored in blue and orange, respectively. Aromatic ring of Phe20 is rotated slightly to stack with the -2A base. Arg35 is pulled toward the RNA molecule and interacts with the 2'-hydroxyl group of -3U. The side chains of His29, Arg28 and Ser34 undergoes local structural changes upon the binding to RNA. **(d)** Close-up view of (a) for the RNA binding site of the C-terminal zinc finger (ZF2, 143-170). The side chains of RNA-bound of U2AF1 ZF2 are presented in green. Aromatic ring of Phe165 is rotated to stack with the -1G base.

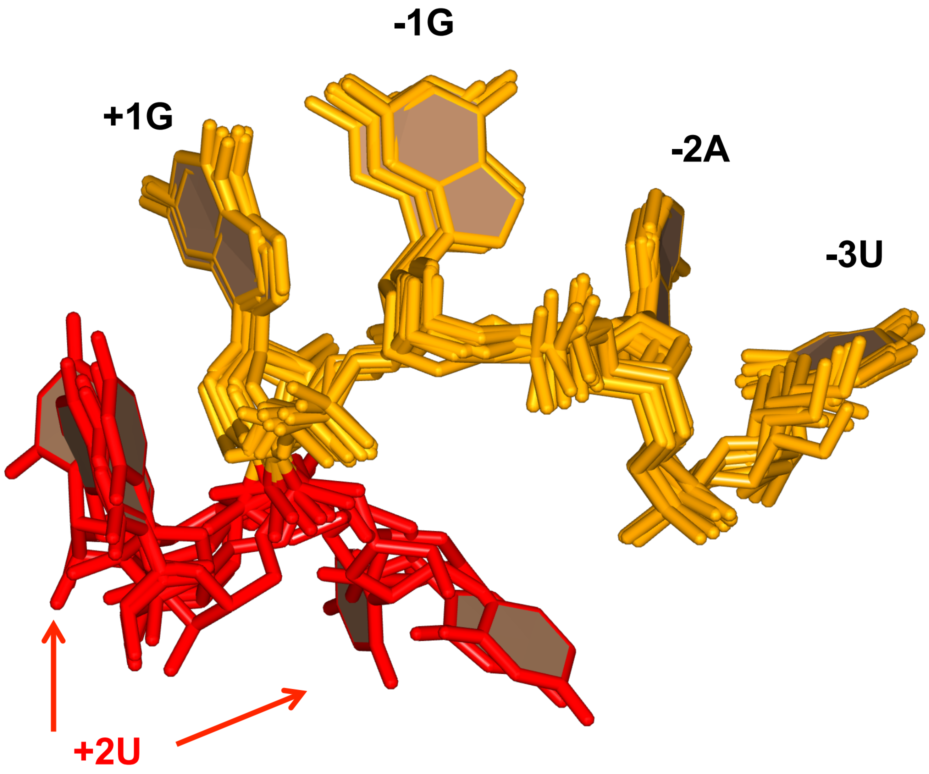

Supplementary Fig. 3

Superposition of nine RNA molecules in the crystal structure.

Structural alignment for nine RNA molecules was carried out automatically with SSM (Secondary Structure Matching) through *COOT* [4]. Nine RNA molecules overlap closely in first four bases of the UAGGU sequences (from -3U to +1G). In contrast, last +2U base shows largely different configuration in the crystal packing. The nucleotide sequence at +2 position could not be strictly recognized by U2AF1.

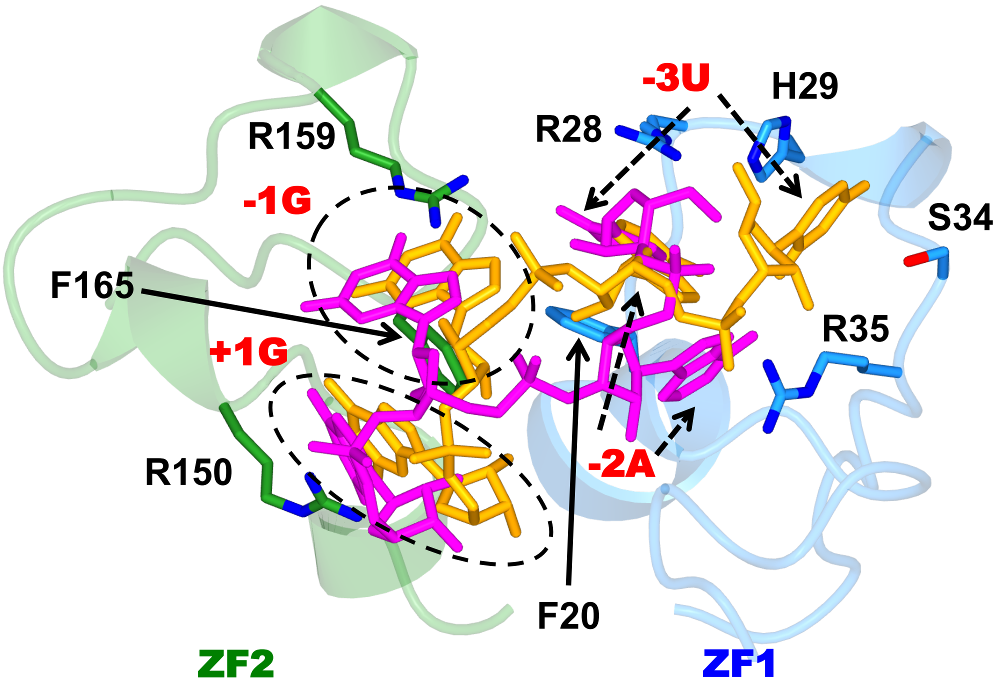

Supplementary Fig. 4

Difference of the RNA structure between the U2AF1-RNA complex (present work) and the modeled structure based on the RNA-free form of U2AF1.

The modeled protein/RNA complex structure was reported on the basis of RNA-free structure in 2015 [3]. RNA molecules are shown in stick representation colored in orange for the present crystal structure of RNA bound-form and in magenta for the RNA binding model reported before, respectively. Normally, in the CCCH-type Zn finger domain, the RNA bases are accommodated between the aromatic ring and positively-charged amino-acid residue [5,6]. Correspondingly, the RNA bases of -1G and +1G are located between Phe and Arg residues on the ZF2 in the crystal structure and they were deduced properly in the model structure. On the other hand, in the novel type of ZF1, the space between Phe20 and Arg35 is not utilized for the RNA base accommodation in the crystal structure. Consequently, the positions of the -2A and -3U bases are different between the present crystal structure and the modeled structure.

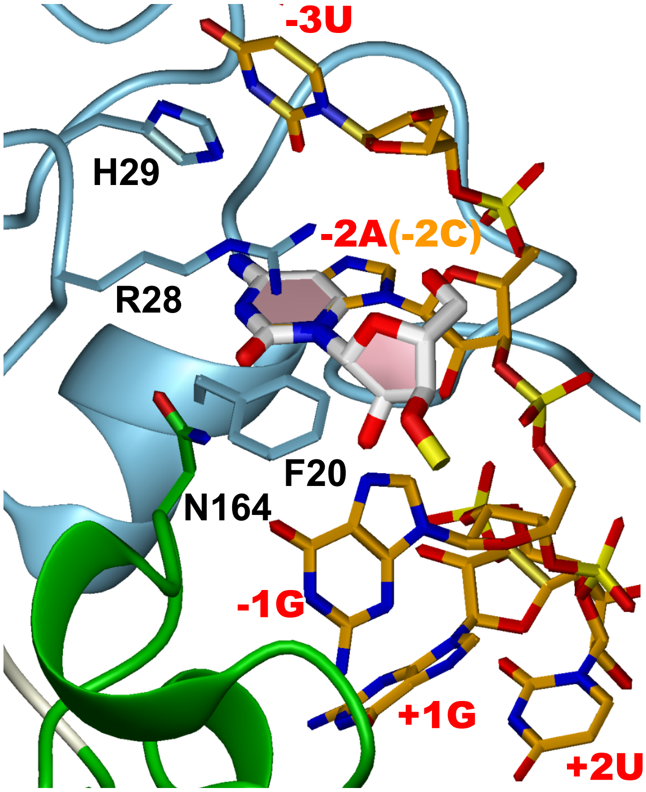

Supplementary Fig. 5

Putative position of the cytidine residue at the -2 position on ZF2.

The Close-up view of the U2AF1 and 5′-UAGGU complex, showing the region around the -2A binding site. The protein is represented by ribbon model colored by sky blue (ZF1), beige (UHM), and green ribbon (ZF2). The bound RNA is shown in stick representation colored with orange (carbon atoms), blue (nitrogen atoms), red (oxygen atoms) and yellow (phosphors atoms). The hypothetical cytosine residue is represented in the anti-configuration as the amino group and the hydrogen-acceptor nitrogen atom of RNA base are overlapped with the corresponding atoms of the -2A. The cytosine residue is shown in thicker stick representation with pink tiles and the bonds are colored with white (carbon atoms), blue (nitrogen atoms), red (oxygen atoms) and yellow (phosphors atoms).

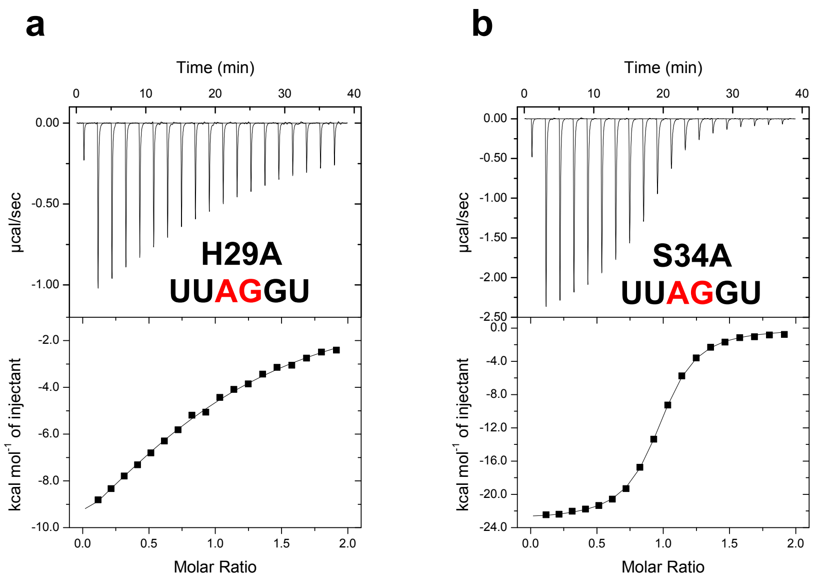

c

Protein	RNA 5' to 3'	N	$-T\Delta S$ (kcal/mol)	ΔH (kcal/mol)	*K*d (µM)
H29A	UUAGGU	1.0 ± 0.1	10.1 ± 3.0	-16.2 ± 2.9	37.5 ± 8.8
S34A	UUAGGU	1.0 ± 0.0	14.4 ± 0.5	-22.6 ± 0.5	0.97 ± 0.04

Supplementary Fig. 6

ITC experiments for the U2AF1 mutants with the RNA fragments.

Raw data of ITC measurements for the H29A mutant of U2AF1 with UUAGGU **(a),** the S34A mutant with UUAGGU **(b)**, and the S34A mutant with UAAGGU **(c)**. The upper panels show the heat release caused by injections of the U2AF complex into RNA. The lower panels show the fitted binding parameters; the solid line in each lower panel represents a calculated curve using the best fit parameters obtained by a nonlinear least-squares fit. **(d)** RNA binding activities calculated by ITC measurements.

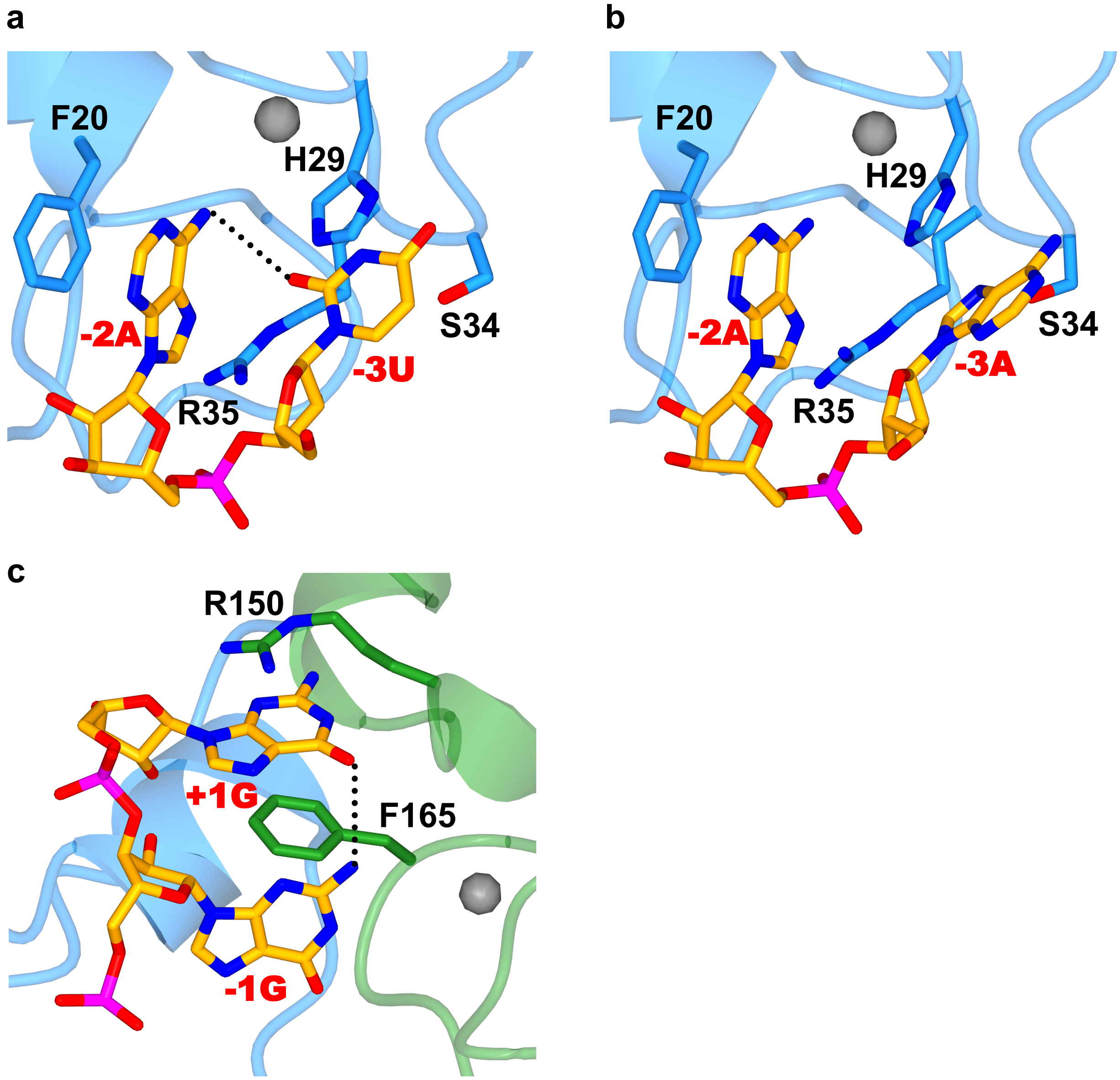

Supplementary Fig. 7

Specific interaction between RNA bases.

Structural interfaces around the -1G and +1G bases in the U2AF1 and 5'-UAGGU complex **(a)**, around the -3A and -2A bases in the U2AF1 and 5'-UAGGU complex **(b)**, and around the -3A and -2A bases in the U2AF1 and 5'-AAGGU complex **(c)**. As shown in (a), N^6-amino group of -1G base makes hydrogen bonding to O^4-hydroxyl group of +1G to stabilize each other. In (b), O^2-hydroxyl group of -3U base makes hydrogen bonding to -2A to stabilize each other. On the other hand, there are no hydrogen bond between -3A and -2A bases in (c). Thus, the -2A and -3A bases in (c) apart farther than the -2A and -3U bases in (b).

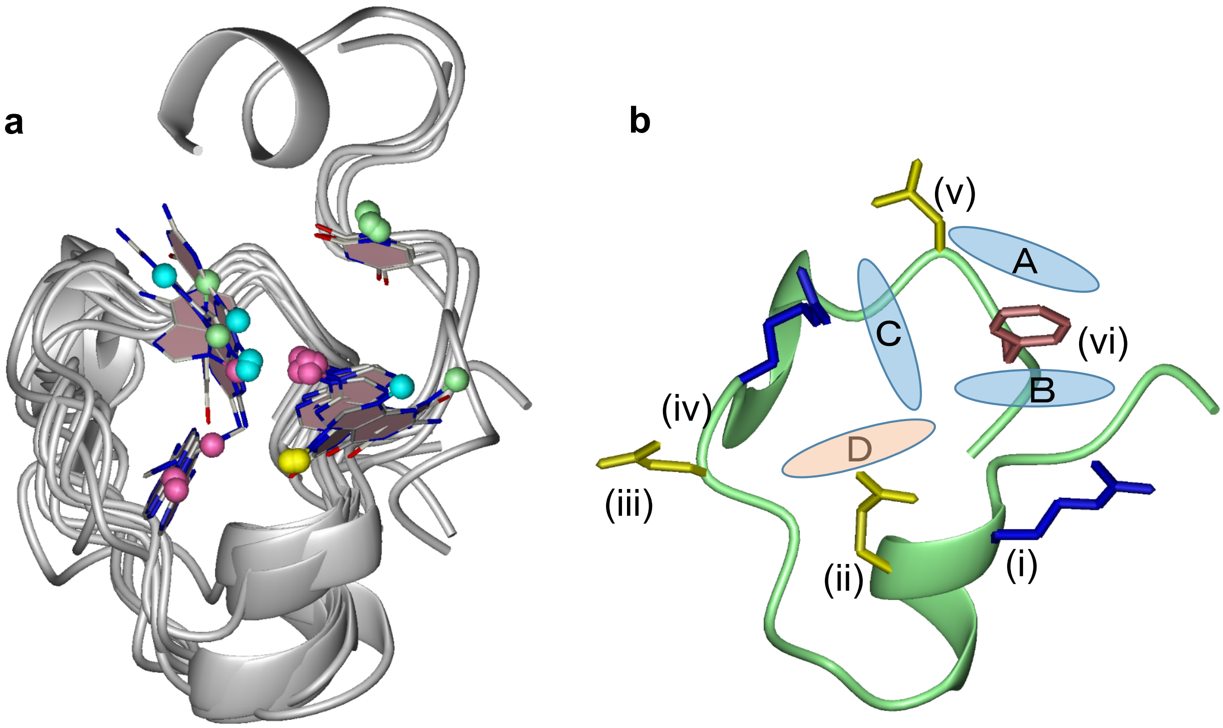

Supplementary Fig. 8

RNA binding pockets on CCCH-type zinc finger domains.

(a) Overlay of ZF domains (MBNL1 ZF3 with 5′-GC/PDBID:3D2S, MBNL1 ZF2 with 5′-GC/PDBID:5U9B, TISIId ZF1 with 5′-UAUU/PDBID:1RGO, mouse Unkempt ZF3 with 5′-UUA/PDBID:5ELH, mouse Unkempt ZF6 with 5′-UAG/PDBID:5ELK, *C. thermophilum* Nab2 ZF5 with 5′-AA/PDBID:4LJ0, CPSF30 ZF2 and ZF3 with 5′-AA/PDBID:6FUW, U2AF1 ZF1 with 5′-UAG /present work, and U2AF1 ZF2 with 5′-GG /present work) with corresponding bound RNA bases [5-10]. ZF domains are represented by gray ribbons. The bound RNA bases are shown by sticks with colored tiles. The tiles and the C1′ carbon atoms of adenine, cytosine, guanine, and uracil bases are colored by pink, yellow, cyan and pale green, respectively. These structures are overlaid manually as the recognized atoms of the nucleotides base could overlap with each other. **(b)** The RNA base recognition sites based on the overlapped nucleotide base shown in (a). The base accommodation sites are shown by colored ellipses (zones A, B, C, and D) on the ternary structure of U2AF1 ZF2. The side chains of the six amino-acid residues, which are responsible for the formation of zones A, B, C and D, are shown by stick and are labeled with (i), (ii), (iii) (iv), (v) and (vi). In the case of U2AF1 ZF2, R150, Q151, Q158, R159, N164 and F165 corresponds to these labeled amino-acid residues, respectively.

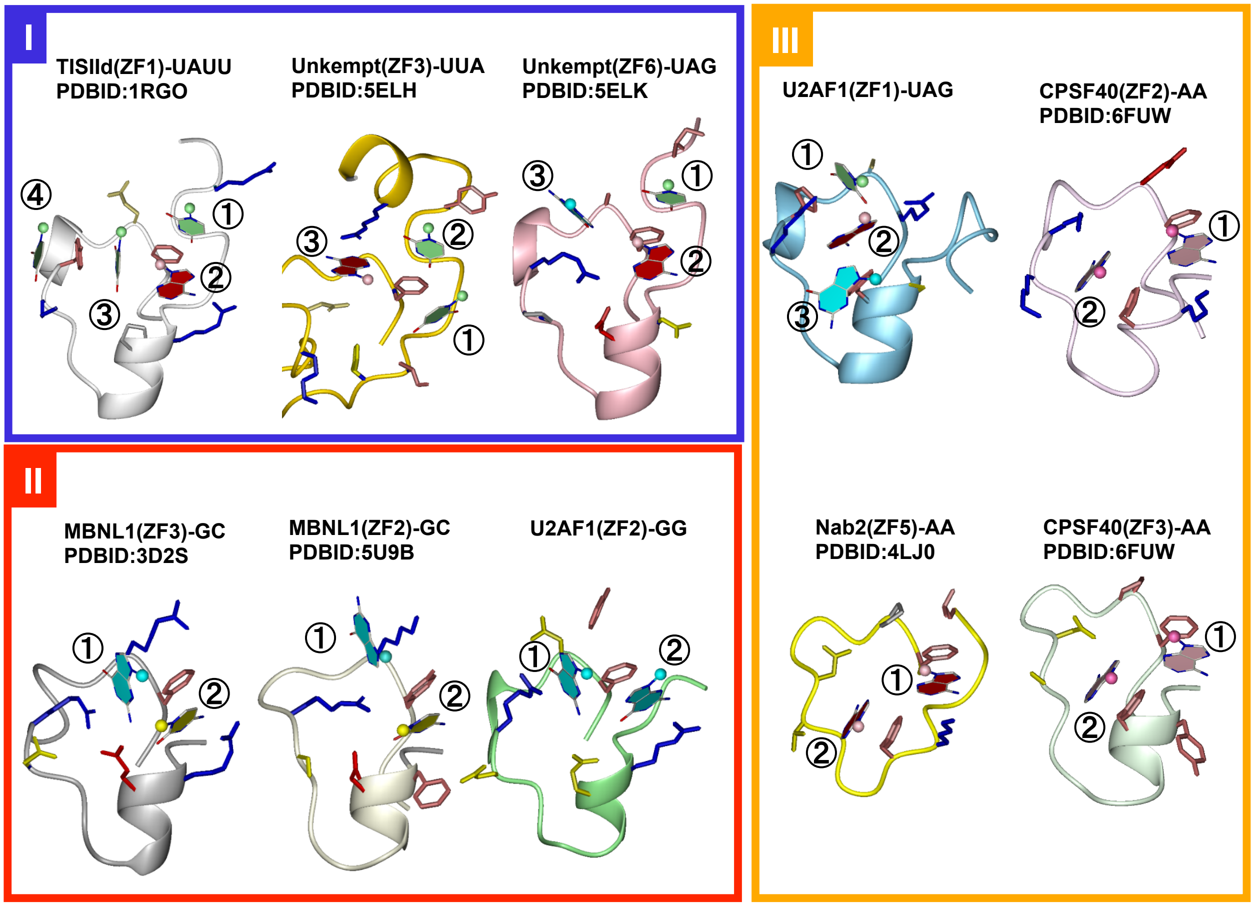

Supplementary Fig. 9

Classification of RNA recognition mode of CCCH-type zinc finger domains.

Classification of the CCCH-type zinc finger domains (annotated as zfCCCH1 and zfCCCH2 (Nab-type) in Pfam32.0 data base) that were overlaid in (a). The members without aromatic amino-acid residue at (iv) are classified into group I and group II. Group I contains the members that do not utilized zone A. and Group II members utilize zone A. Group III contains the members with aromatic amino-acid residue at (iv) position. U2AF1 ZF1 is classified in Group III. Previously determined complex structure of Group III members contains aromatic amino-acid residues both at (ii) and (vi) positions. However, U2AF1 ZF1 has Arg residues at (vi) instead of the canonical aromatic amino-acid residue. RNA bases are shown according to (a) and the positions from the 5′-end are shown by numbers in circles.

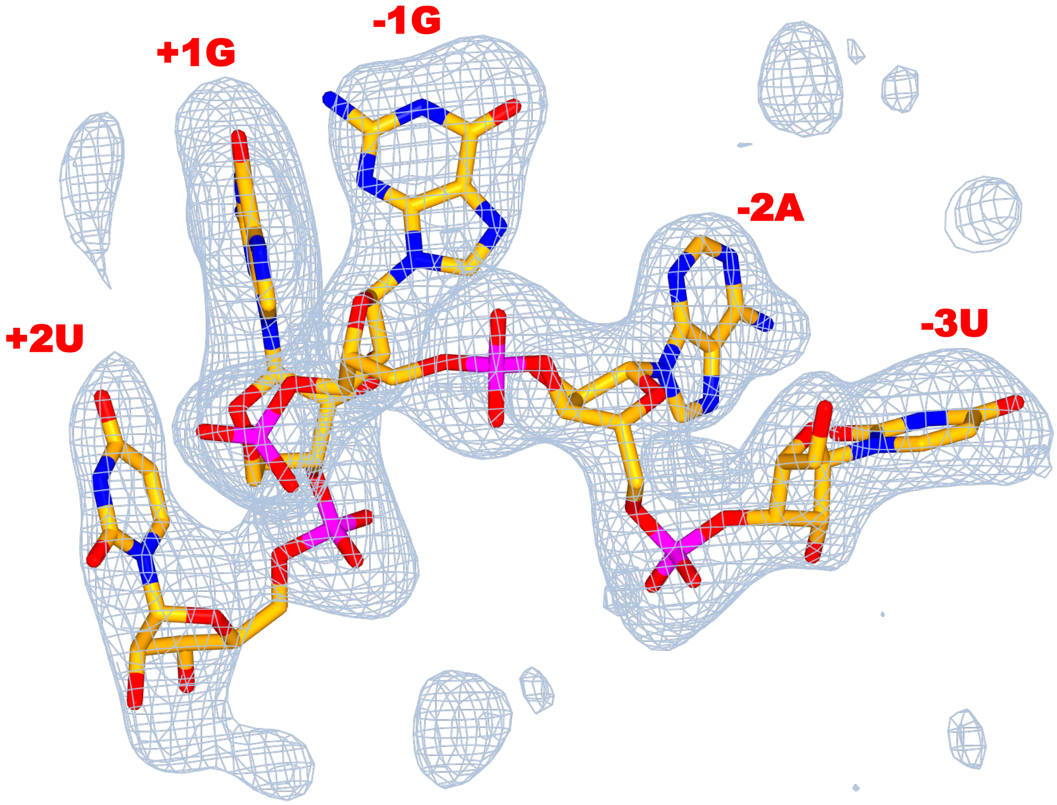

Supplementary Fig. 10

Electron density map of RNA.

Representative *mFo-DFc* omit map around the RNA (grey), contoured at 3.0 sigma. The weighted *mFo-DF*c omit map was calculated by omitting selected RNA from the final model, using the program of *Polder Maps* in *Phenix* suite[11].

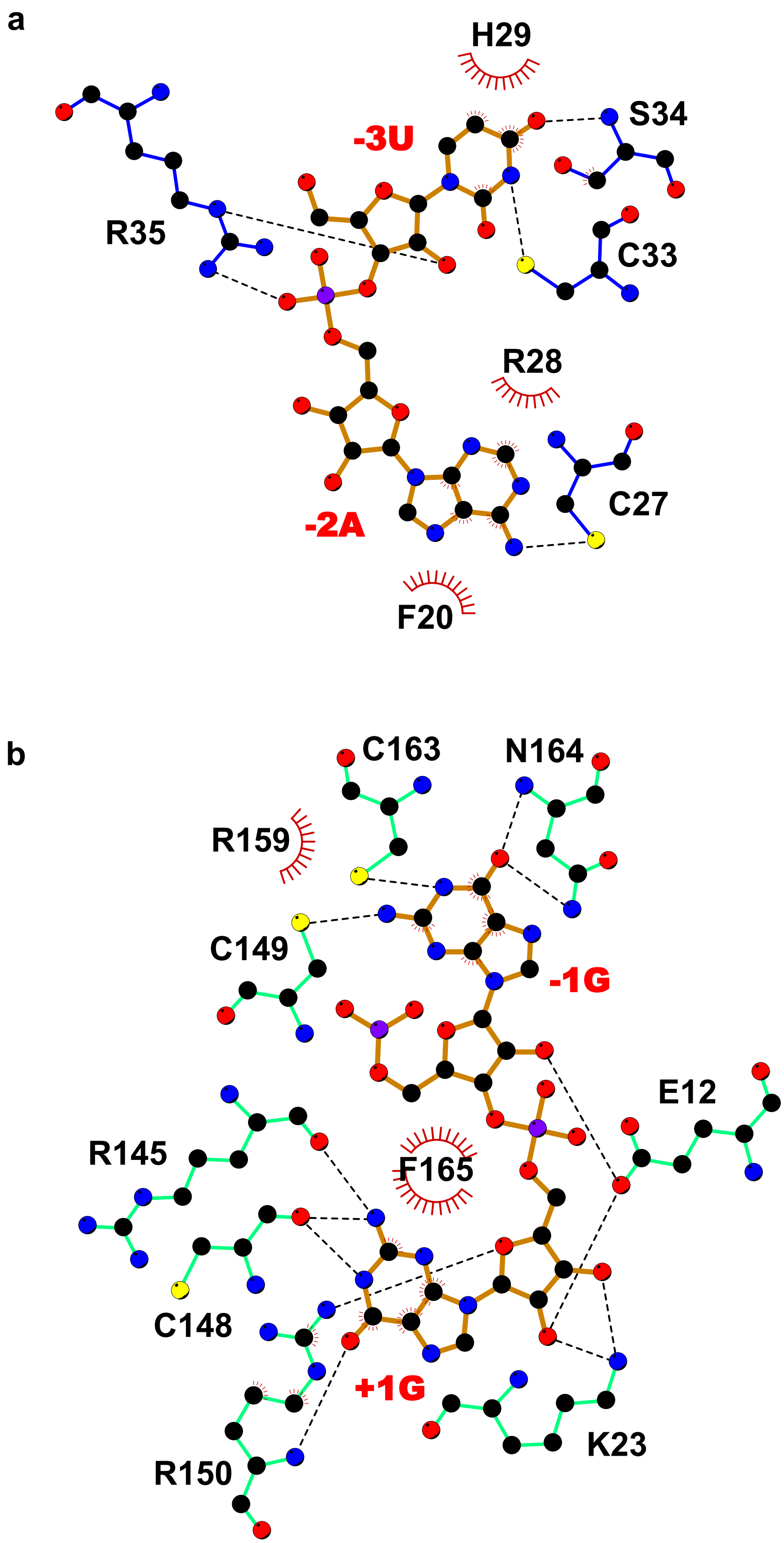
a
H29
S34
-3U
R35
C33
R28
C27
-2A
F20
b
C163
N164
R159
C149
-1G
E12
R145
F165
C148
+1G
K23
R150

Supplementary Fig. 11

Schematic diagram showing the protein-RNA interactions.

(a) Interactions of -3U and -2A bases of RNA on the ZF1, and **(b)** interactions of -1G and +1G bases of RNA on the ZF2. The hydrophobic interactions between RNA and the protein (distance, 3.5-3.9 Å) are indicated with dotted arcs. Hydrogen bonds (2.3-3.5 Å) are shown as dotted lines. The figures were rendered with LIGPLOT [12].

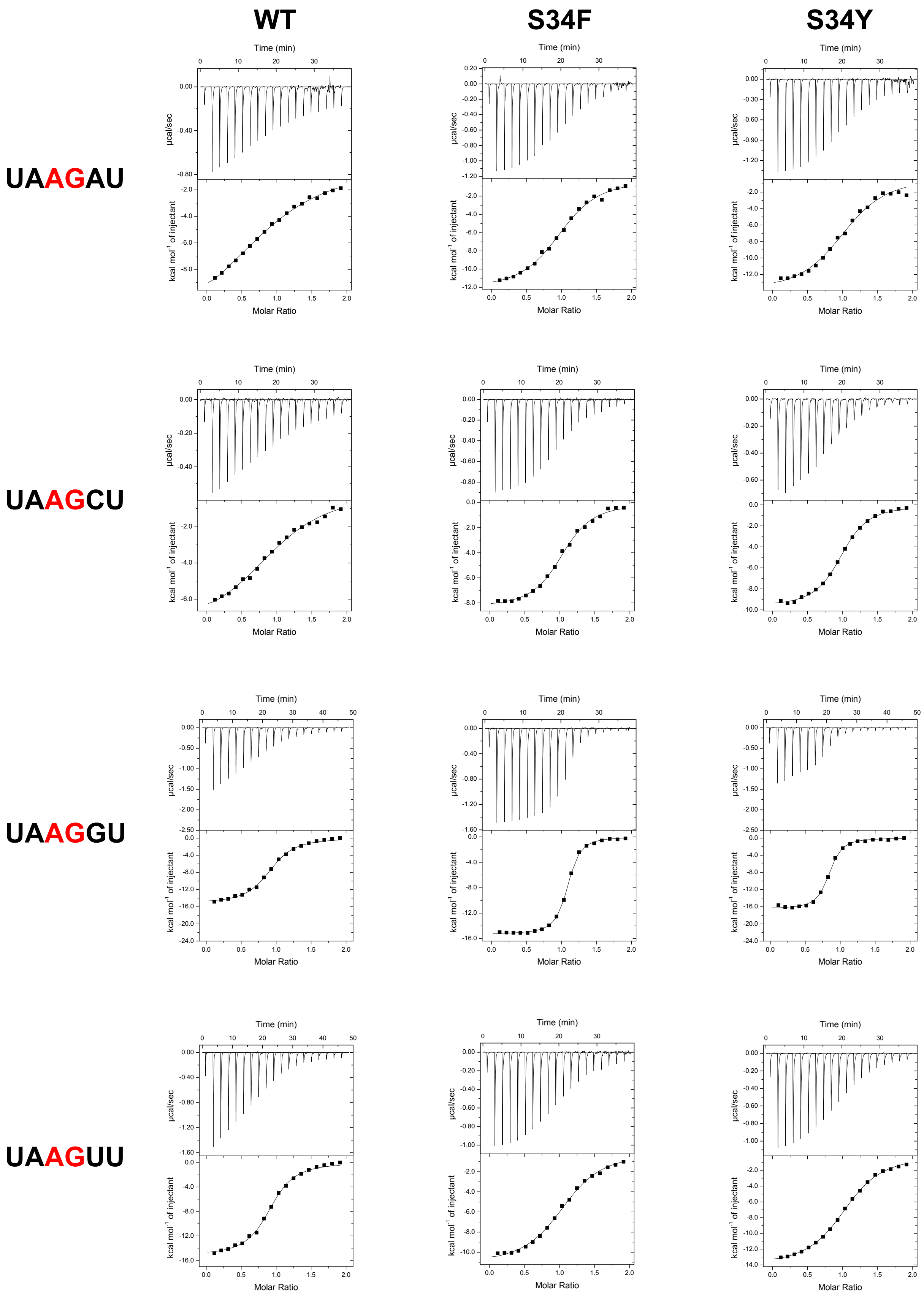
WT
S34F
S34Y
UAAGAU
UAAGCU
UAAGGU
UAAGUU
Time (min)
µcal/sec
kcal mol-1 of injectant
Molar Ratio

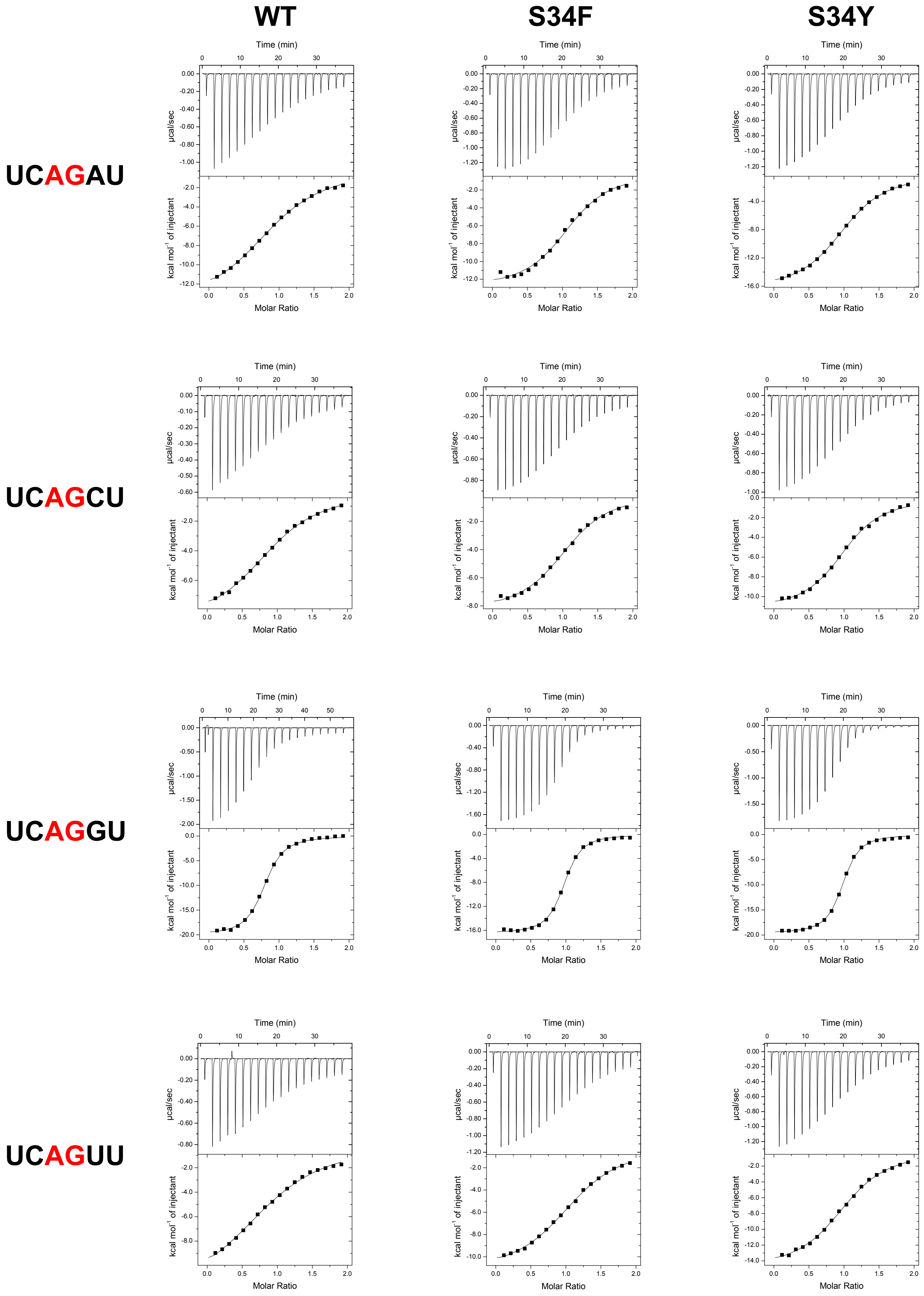
WT
S34F
S34Y
UCAGAU
UCAGCU
UCAGGU
UCAGUU
Time (min)
µcal/sec
kcal mol⁻¹ of injectant
Molar Ratio

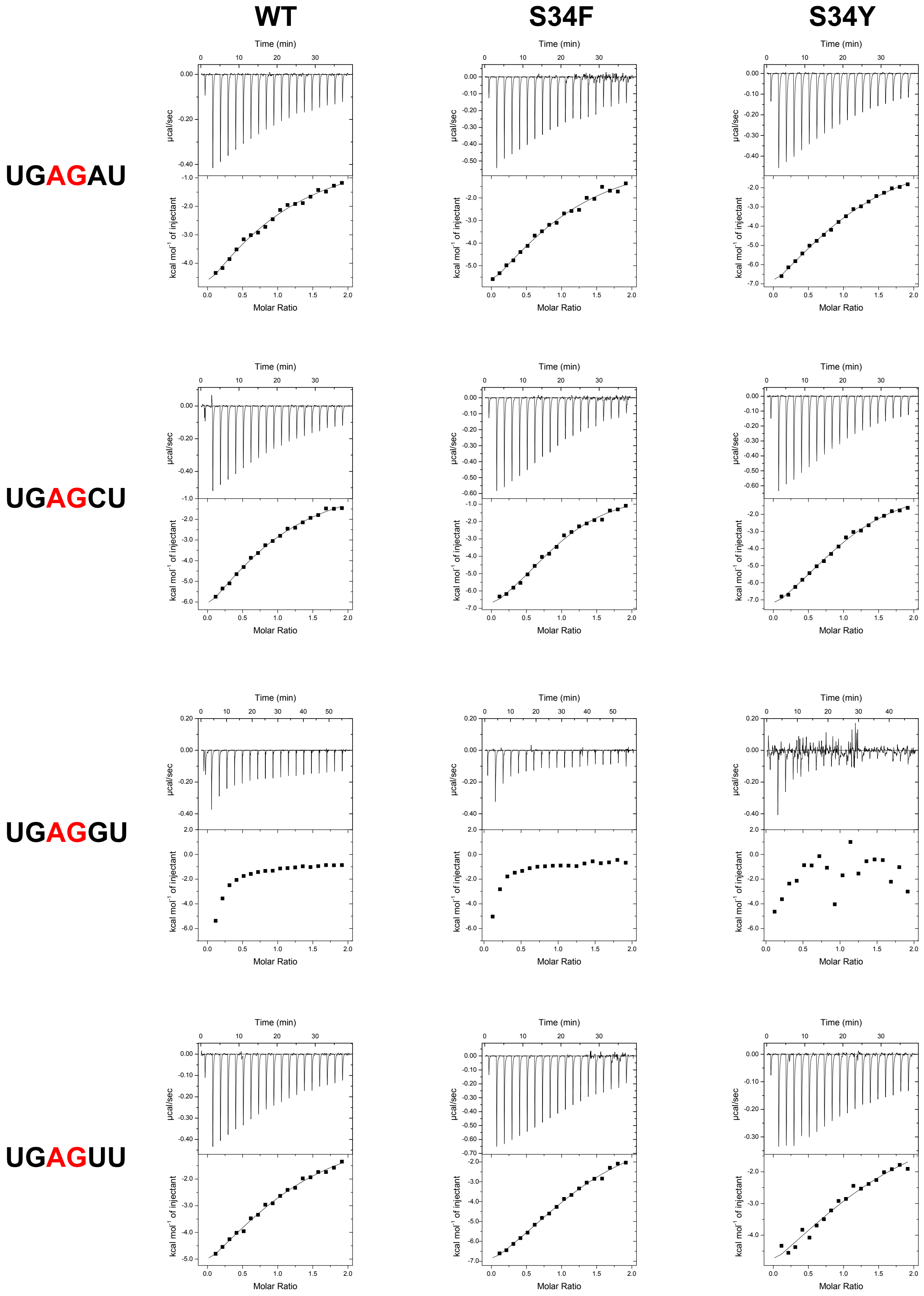

WT
S34F
S34Y
UGAGAU
UGAGCU
UGAGGU
UGAGUU
Time (min)
µcal/sec
kcal mol-1 of injectant
Molar Ratio

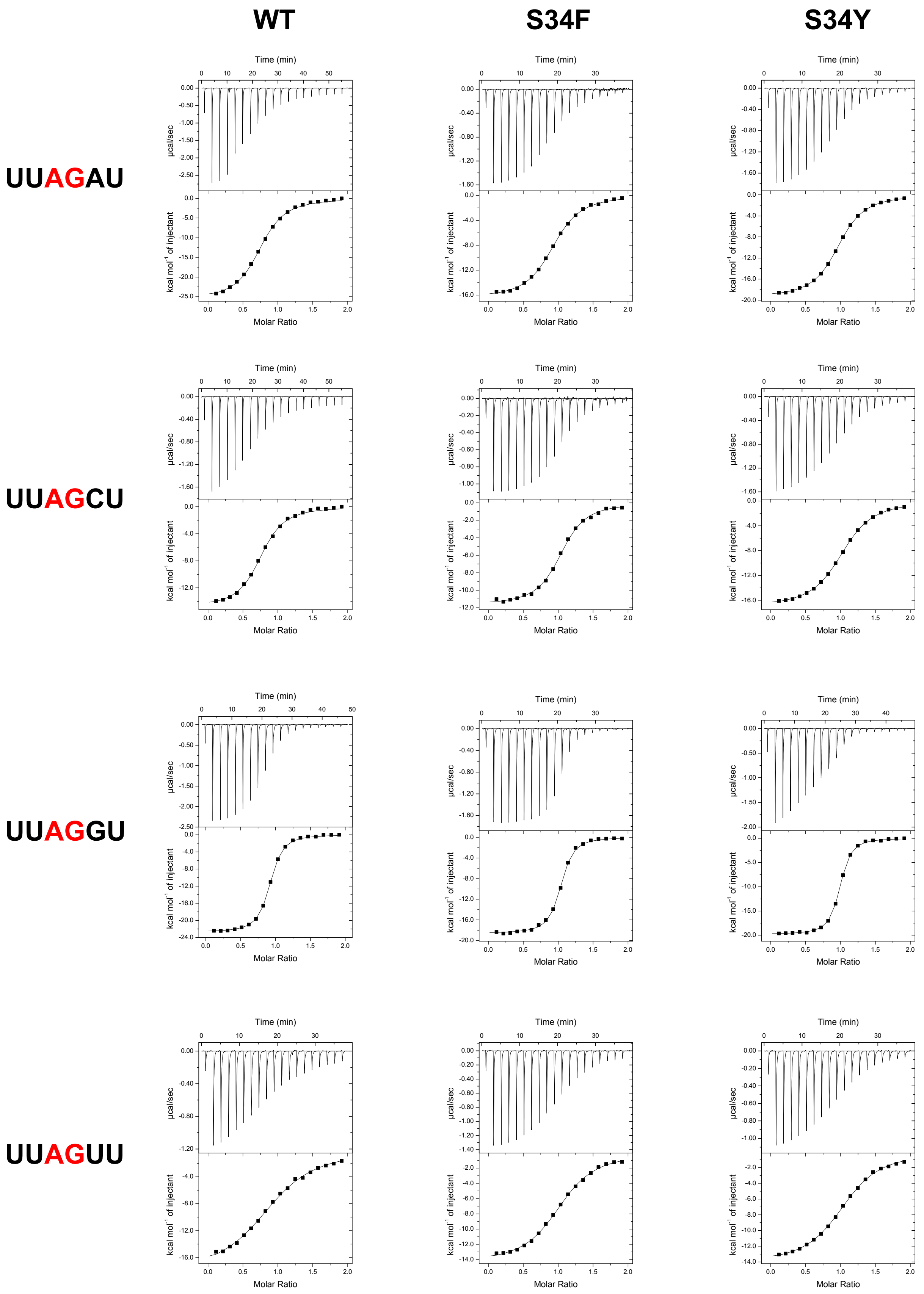
WT
S34F
S34Y
UUAGAU
UUAGCU
UUAGGU
UUAGUU
Time (min)
µcal/sec
kcal mol-1 of injectant
Molar Ratio

Supplementary Fig. 12

Raw data of ITC measurements.

The upper panels show the heat release caused by injections of the U2AF complex into RNA. The lower panels show the fitted binding parameters; the solid line in each lower panel represents a calculated curve using the best fit parameters obtained by a nonlinear least-squares fit.

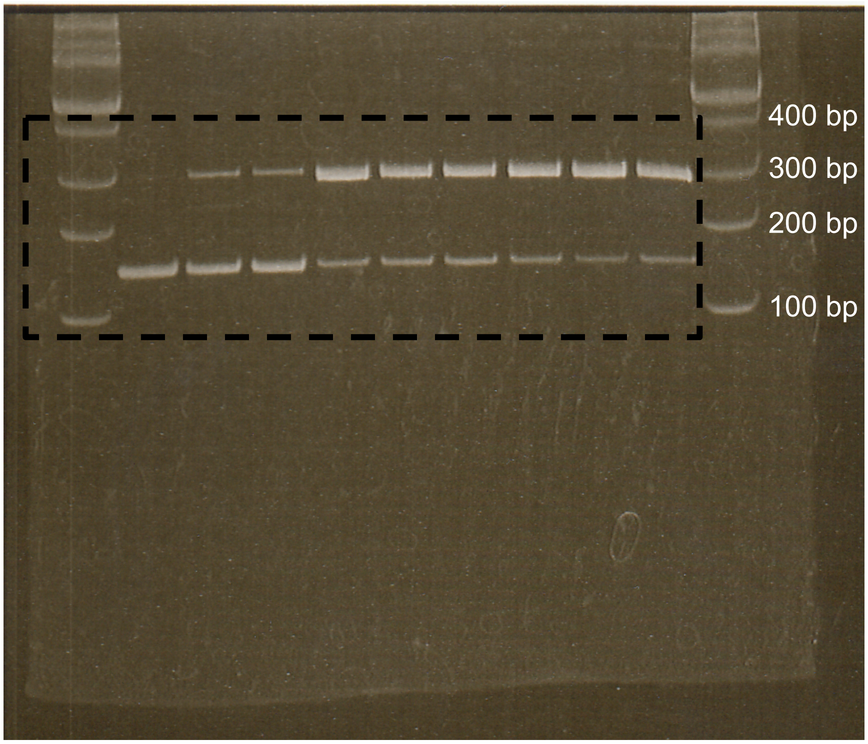

Supplementary Fig. 13

Raw data of mini gene splicing assay.

Raw image of gel electrophoresis results from RT-PCR products of mini gene splicing assay. Molecular weight markers are shown in right and left side lanes. The part of image enclosed with dashed line is shown in Fig. 5b.

Supplementary Table 1

RNA binding activities to the U2AF1 WT evaluated by ITC measurement

	**WT**		
RNA 5' to 3'	**N**	**-*T*Δ*S*** (kcal/mol)	**Δ*H*** (kcal/mol)
UAAGAU	1.0 ± 0.1	7.7 ± 2.8	-14.2 ± 2.9
UAAGCU	0.9 ± 0.2	2.1 ± 1.4	-8.7 ± 1.4
UAAGGU	0.9 ± 0.0	7.4 ± 0.4	-15.3 ± 0.3
UAAGUU	0.8 ± 0.0	8.3 ± 2.2	-14.6 ± 2.3
UCAGAU	0.9 ± 0.1	9.6 ± 3.8	-16.3 ± 4.0
UCAGCU	0.9 ± 0.1	3.4 ± 2.2	-10.3 ± 2.2
UCAGGU	0.9 ± 0.1	10.9 ± 2.9	-18.8 ± 2.8
UCAGUU	1.0 ± 0.1	7.8 ± 2.7	-14.3 ± 2.8
UGAGAU	0.9 ± 0.1	8.5 ± 3.4	-14.2 ± 3.3
UGAGCU	0.9 ± 0.1	4.4 ± 1.1	-10.4 ± 1.0
UGAGGU	ND	ND	ND
UGAGUU	1.0 ± 0.3	7.5 ± 7.6	-13.3 ± 7.4
UUAGAU	0.8 ± 0.1	17.4 ± 2.7	-25.2 ± 2.9
UUAGCU	0.9 ± 0.1	7.4 ± 1.6	-14.9 ± 1.8
UUAGGU	0.9 ± 0.0	14.2 ± 0.3	-22.9 ± 0.2
UUAGUU	0.9 ± 0.1	12.5 ± 2.5	-19.5 ± 2.5

Supplementary Table 2

RNA binding activities to the U2AF1 mutants evaluated by ITC measurement

	**S34F**			**S34Y**		
RNA 5' to 3'	**N**	***-TΔS*** (kcal/mol)	***ΔH*** (kcal/mol)	**N**	***-TΔS*** (kcal/mol)	***ΔH*** (kcal/mol)
UAAGAU	1.1 ± 0.0	6.2 ± 2.5	-13.7 ± 2.6	1.1 ± 0.1	8.6 ± 3.0	-13.3 ± 1.5
UAAGCU	1.0 ± 0.1	1.5 ± 1.6	-9.2 ± 1.6	1.0 ± 0.0	1.9 ± 1.0	-9.7 ± 0.8
UAAGGU	1.0 ± 0.1	7.7 ± 2.5	-16.5 ± 2.7	0.8 ± 0.0	8.0 ± 0.3	-16.7 ± 0.2
UAAGUU	1.0 ± 0.1	5.5 ± 2.6	-13.0 ± 2.7	1.1 ± 0.0	5.4 ± 1.9	-13.0 ± 1.6
UCAGAU	1.1 ± 0.1	8.0 ± 3.5	-15.2 ± 3.5	1.1 ± 0.0	8.4 ± 2.1	-15.7 ± 1.8
UCAGCU	1.0 ± 0.1	3.0 ± 2.2	-10.2 ± 2.3	1.1 ± 0.0	3.1 ± 1.9	-10.6 ± 1.6
UCAGGU	0.9 ± 0.1	9.2 ± 2.2	-17.6 ± 2.5	1.0 ± 0.0	10.4 ± 1.2	-18.9 ± 1.1
UCAGUU	1.1 ± 0.1	6.3 ± 3.1	-13.4 ± 3.2	1.1 ± 0.0	6.9 ± 2.2	-14.1 ± 2.0
UGAGAU	1.0 ± 0.1	5.8 ± 5.7	-12.0 ± 5.6	1.1 ± 0.0	5.7 ± 0.8	-11.8 ± 0.7
UGAGCU	1.0 ± 0.1	3.1 ± 2.6	-9.7 ± 2.4	1.1 ± 0.0	3.9 ± 0.2	-10.1 ± 0.1
UGAGGU	ND	ND	ND	ND	ND	ND
UGAGUU	1.2 ± 0.3	6.1 ± 3.6	-12.2 ± 3.6	1.3 ± 0.2	2.7 ± 1.5	-8.7 ± 1.5
UUAGAU	0.9 ± 0.1	10.6 ± 2.8	-18.4 ± 3.0	1.1 ± 0.1	9.9 ± 1.6	-17.7 ± 2.0
UUAGCU	1.0 ± 0.1	4.8 ± 1.8	-12.8 ± 1.9	1.1 ± 0.0	7.5 ± 3.5	-15.4 ± 3.1
UUAGGU	1.0 ± 0.0	9.5 ± 0.4	-18.4 ± 0.5	0.9 ± 0.0	10.6 ± 0.3	-19.5 ± 0.3
UUAGUU	1.0 ± 0.1	8.3 ± 2.5	-15.9 ± 2.7	1.1 ± 0.0	5.7 ± 0.9	-13.7 ± 0.9

Supplementary References

1. Sievers, F. *et al.* Fast, scalable generation of high-quality protein multiple sequence alignments using Clustal Omega. *Mol. Syst. Biol.* **7,** 539 (2011).
2. Robert, X. & Gouet, P. Deciphering key features in protein structures with the new ENDscript server. *Nucleic Acids Res.* **42,** W320–4 (2014).
3. Yoshida, H. *et al.* A novel 3' splice site recognition by the two zinc fingers in the U2AF small subunit. *Genes Dev.* **29,** 1649–1660 (2015).
4. Emsley, P., Lohkamp, B., Scott, W. G. & Cowtan, K. Features and development of Coot. *Acta Crystallogr. D Biol. Crystallogr.* **66,** 486–501 (2010).
5. Teplova, M. & Patel, D. J. Structural insights into RNA recognition by the alternative-splicing regulator muscleblind-like MBNL1. *Nat. Struct. Mol. Biol.* **15,** 1343–1351 (2008).
6. Hudson, B. P., Martinez-Yamout, M. A., Dyson, H. J. & Wright, P. E. Recognition of the mRNA AU-rich element by the zinc finger domain of TIS11d. *Nat. Struct. Mol. Biol.* **11,** 257–264 (2004).
7. Park, S. *et al.* Structural Basis for Interaction of the Tandem Zinc Finger Domains of Human Muscleblind with Cognate RNA from Human Cardiac Troponin T. *Biochemistry* **56,** 4154–4168 (2017).
8. Murn, J., Teplova, M., Zarnack, K., Shi, Y. & Patel, D. J. Recognition of distinct RNA motifs by the clustered CCCH zinc fingers of neuronal protein Unkempt. *Nat. Struct. Mol. Biol.* **23,** 16–23 (2016).
9. Clerici, M., Faini, M., Muckenfuss, L. M., Aebersold, R. & Jinek, M. Structural basis of AAUAAA polyadenylation signal recognition by the human CPSF complex. *Nat. Struct. Mol. Biol.* **25,** 135–138 (2018).
10. Kuhlmann, S. I., Valkov, E. & Stewart, M. Structural basis for the molecular recognition of polyadenosine RNA by Nab2 Zn fingers. *Nucleic Acids Res.* **42,** 672–680 (2014).
11. Liebschner, D. *et al.* Polder maps: improving OMIT maps by excluding bulk solvent. *Acta Crystallogr D Struct Biol* **73,** 148–157 (2017).
12. Laskowski, R. A. & Swindells, M. B. LigPlot+: multiple ligand-protein interaction diagrams for drug discovery. *J Chem Inf Model* **51,** 2778–2786 (2011).
